# Neuroprotective Properties of Mildronate, a Small Molecule, in a Rat Model of Parkinson’s Disease

**DOI:** 10.3390/ijms11114465

**Published:** 2010-11-09

**Authors:** Vija Z. Klusa, Sergejs Isajevs, Darja Svirina, Jolanta Pupure, Ulrika Beitnere, Juris Rumaks, Simons Svirskis, Baiba Jansone, Zane Dzirkale, Ruta Muceniece, Ivars Kalvinsh, Harry V. Vinters

**Affiliations:** 1 Department of Pharmacology, Faculty of Medicine, University of Latvia, Riga, Latvia; 2 Department of Pathology, Faculty of Medicine, University of Latvia, Riga, Latvia; 3 Latvian Institute of Organic Synthesis, Riga, Latvia; 4 Department of Pathology and Laboratory of Medicine and Neurology, University of California Los Angeles, Los Angeles, CA, USA

**Keywords:** Parkinson’s disease, 6-OHDA model, neuroimmunological biomarkers, mildronate, small molecule

## Abstract

Previously, we have found that mildronate [3-(2,2,2-trimethylhydrazinium) propionate dihydrate], a small molecule with charged nitrogen and oxygen atoms, protects mitochondrial metabolism that is altered by inhibitors of complex I and has neuroprotective effects in an azidothymidine-neurotoxicity mouse model. In the present study, we investigated the effects of mildronate in a rat model of Parkinson’s disease (PD) that was generated via a unilateral intrastriatal injection of the neurotoxin 6-hydroxydopamine (6-OHDA). We assessed the expression of cell biomarkers that are involved in signaling cascades and provide neural and glial integration: the neuronal marker TH (tyrosine hydroxylase); ubiquitin (a regulatory peptide involved in the ubiquitin-proteasome degradation system); Notch-3 (a marker of progenitor cells); IBA-1 (a marker of microglial cells); glial fibrillary acidic protein, GFAP (a marker of astrocytes); and inducible nitric oxide synthase, iNOS (a marker of inflammation). The data show that in the 6-OHDA-lesioned striatum, mildronate completely prevented the loss of TH, stimulated Notch-3 expression and decreased the expression of ubiquitin, GFAP and iNOS. These results provide evidence for the ability of mildronate to control the expression of an array of cellular proteins and, thus, impart multi-faceted homeostatic mechanisms in neurons and glial cells in a rat model of PD. We suggest that the use of mildronate provides a protective effect during the early stages of PD that can delay or halt the progression of this neurodegenerative disease.

## 1. Introduction

Parkinson’s disease (PD) is clearly defined as a neurodegenerative disorder that involves multifactorial mechanisms, such as mitochondrial dysfunction, neuronal apoptosis, neuroinflammation and microglial activation, which leads to the progressive loss of dopamine-producing cells in the nigro-striatal system [1,2]. Unfortunately, the conventional therapy for PD focuses primarily on the consequences of this neuronal loss: the stimulation of degenerating dopamine cells [3]. Therefore, novel pharmacotherapeutic strategies are being developed that focus on cellular targets to prevent or delay dopamine-induced cell death at much earlier stages of neurodegeneration. These approaches may reveal novel strategies for the treatment of PD.

Mitochondria can be considered one of these novel drug targets. There are several arguments supporting the view that genetic defects in mitochondria play a prevalent role in the pathogenesis of PD [4–6]. Defects in complex I are considered a central and early cause of PD [4] because a deficiency in complex I activity initiates further respiratory chain dysfunctions that involve mitochondria-dependent apoptotic cascades and result in cell death [7].

Therefore, the protection of mitochondria, even their repair mechanisms at the level of complex I, may be a key strategy in limiting mitochondrial damage and ensuring cellular integrity [8]. At present, drugs that provide such effect have not been found.

A more complicated problem is the determination of ways to control the development of pathological changes in protein conformations that are associated with PD. Mutations in the genes that encode a series of proteins, particularly α-synuclein (SNCA), parkin, DJ-1, PINK1, LRRK2 and HTR2A, lead to the formation of misfolded protein conformations that are harmful to cells [1,2]. The aggregation of α-synuclein in Lewy bodies plays a crucial role in cell death because such aggregates activate pro-inflammatory molecules and switch on the neuroinflammatory and apoptotic events that lead to cell death. Recent evidence suggests that misfolded proteins and their aggregates are closely related to the activities of endogenous or molecular chaperones, which are intracellular molecules that are capable of unfolding proteins [9]. Among these chaperones, the heat shock protein (HSP) family is the most commonly studied group.

Decreasing levels of HSP and α-synuclein aggregation in Lewy bodies coincide with the progression of PD [10]. Experimental data show that HSP70 may inhibit α-synuclein fibril formation [11] and possess anti-apoptotic activity [10].

Designing chemical or pharmacological chaperones (pharmacoperons) that can unfold misfolded proteins in a similar manner to endogenous molecules is an attractive approach for future PD therapies [12]. Numerous molecules have been shown to possess the properties of chemical chaperones, such as glycerol, diltiazem, vinblastine, trimethylamine *N*-oxide, rifampicin, flavonoids and glycine betaine [13–17].

A very important consequence of a build-up and aggregation of misfolded proteins is the impairment of the ubiquitin-proteasome degradation system (UPS) [10]. Normally, proteins that are conjugated to ubiquitin are targeted to the UPS where they undergo proteolytic degradation [18]. The ubiquitin/proteasome pathway is the major proteolytic quality control system in cells, and ubiquitin-enriched Lewy bodies in dopaminergic neurons are a hallmark of PD [19]. A high level of ubiquitin and ubiquitinated proteins (*i.e*., ubiquitin that is covalently attached to target proteins) in Lewy bodies indicates that protein degradation by proteasomes is impaired [20]. Therefore, ubiquitination pathways may play a central role in the pathogenesis of PD [18], and the regulation of the level of ubiquitin may serve as another drug target.

Others studies emphasize glial cells as a target for the treatment of PD. Microglial activation plays an important role in the early stages (before dopaminergic cell death) of PD pathogenesis [21]. This activation triggers the onset of a molecular cascade that leads to the progressive degeneration of cells [22]. Therefore, the blockade of microglial activation might be a target for therapeutic intervention in PD [23]. Moreover, a new concept of glia as a stem cell element in the brain has been recently proposed [24]. Furthermore, astrocytes are involved in the formation of synapses and in the modulation of synaptic functions via a bidirectional communication with neurons [25]. Brain damage caused by stroke, degeneration or demyelination may elicit reactive astrogliosis, which has been suggested to impede neuronal survival [23]. Conversely, activated astrocytes may promote neurogenesis by producing energy-producing compounds and neurotrophins [26,27]. Neurotrophins, such as glial cell line-derived neurotrophic factor (GDNF) [28] and mesencephalic astrocyte-derived neurotrophic factor (MANF) [29], have been proposed to promote the survival and differentiation of dopamine neurons. Nevertheless, their clinical use is limited by their large molecular size and antigenicity.

In this context, the idea of adult neurogenesis as a potential tool for the restoration of lost dopamine neurons might open new vistas for the development of novel therapeutics in PD [30]. Although neural or fetal stem cell transplantation has been used previously to replace damaged neurons [31], the stimulation of endogenous progenitor cells is an exciting alternative [32]. In PD brain structures, a decrease in progenitor cell proliferation has been demonstrated [33]. In a 6-OHDA-lesioned PD model in rats, an increase in progenitor cell proliferation in nigro-striatal structures has been noted [32]. The search for drugs that are capable of regulating progenitor cell proliferation in the treatment of PD is a very new and critical issue. Guanosine was recently shown to induce the proliferation of neural progenitor stem cells in the adult SN and subventricular zone in a proteasome inhibitor-induced PD model in rats [34].

Small molecules that are capable of altering the structure of proteins may be optimal pharmacophores (e.g., positively charged moieties) and serve as privileged molecules that can bind easily to the negatively charged amino acids of cellular proteins and target protein-protein interactions. Therefore, these small molecules may affect protein conformations and act as multivalent regulators of intracellular processes.

Small molecules have been suggested to function as multi-protein co-activators of gene transcription [35]. Therefore, small molecules may demonstrate a beneficial protective effect in PD by slowing or halting the progression of the disease during its early stages.

**Figure 1.**
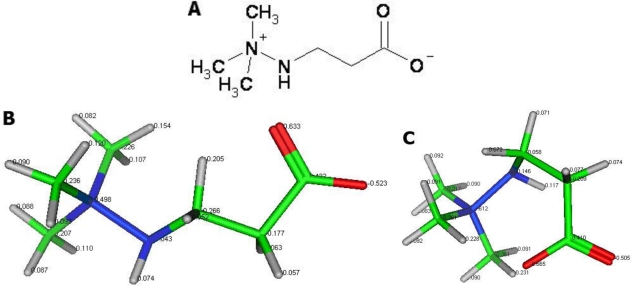
The structure of mildronate (**A**) and models of its linear (**B**) and cyclic (**C**) conformations. Charged atoms in the mildronate structure: nitrogen in blue; oxygen in red; lipophilic hydrogen in gray. When mildronate forms a cyclic conformation, it can easily penetrate the brain and cells.

We propose that mildronate (Figure 1), a representative of the aza-butyrobetaine class of compounds, partially meets the requirements for small molecules. Mildronate possesses a quaternary nitrogen and a negatively charged oxygen. These charged moieties might change the conformation of mildronate from a linear to a cyclic one via intramolecular electrostatic interactions. Cyclic mildronate is a neutral molecule, which allows it to penetrate the brain. Recently, an HPLC method was used to detect mildronate at a concentration of 102 ± 22 nmol/g in brain tissue after a 21 day intraperitoneal administration of 100 mg/kg [36]. In an azidothymidine neurotoxicity model in rats, mildronate suppressed the neuroinflammatory and apoptotic processes in brain tissue by protecting the abnormal expression of cytochrome oxidase c, caspase-3, iNOS, cellular apoptosis susceptibility (CAS) protein and glial fibrillary acidic protein (GFAP) [37]. In addition, data from our previous studies have shown that mildronate plays a protective role against mitochondrial complex I damage caused by mitochondria-compromising substances, such as rotenone and the anti-HIV drug azidothymidine [38].

The present study investigated the effects of mildronate in a 6-OHDA rat model of PD by assessing cellular biomarkers that are involved in signaling cascades and that are crucial for neural and glial integration: the neuronal marker TH (tyrosine hydroxylase); ubiquitin (a regulatory peptide involved in the ubiquitin-proteasome degradation system); Notch-3 (a marker of progenitor cells); IBA-1 (ionised calcium-binding adaptor molecule 1, a marker of microglia); glial fibrillary acidic protein, GFAP (a marker of astrocytes); inducible nitric oxide synthase, iNOS (a marker of inflammation).

## 2. Results

### 2.1. Effects on Apomorphine-Induced Rotations

**Figure 2.**
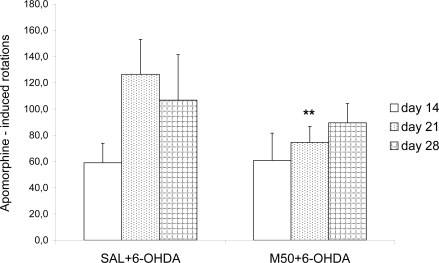
Number of apomorphine-induced contralateral rotations in rats (n = 8 per group). The number of apomorphine-induced contralateral rotations were documented over 30 min on days 14, 21 and 28 after a unilateral intrastriatal injection of 6-OHDA (20 μg) into the right striatum. Apomorphine was administered subcutaneously at a dose of 0.2 mg/kg. Pretreatment with saline (SAL, 1 mL/kg) or mildronate at a dose of 50 mg/kg (M50) was performed via intraperitoneal administration for two weeks before the administration of 6-OHDA (SAL + 6-OHDA and M50 + 6-OHDA), at ** p < 0.01, M50 + 6-OHDA *vs.* SAL + 6-OHDA group, day 21, unpaired t-test.

In saline control and mildronate-treated (50 or 100 mg/kg) rats without 6-OHDA lesions, apomorphine did not induce rotations (data not shown). Significant apomorphine-induced turning rates on days 14, 21 and 28 after 6-OHDA injection were observed, particularly on day 21 (Figure 2). Mildronate (50 mg/kg) in combination with 6-OHDA significantly reduced apomorphine-induced rotations on day 21 after surgery (78.8 ± 13.5 *vs.* 147.0 ± 20.9, p = 0.003); on day 28, a tendency toward an increased rotation was observed. A mildronate dose of 100 mg/kg showed an effect that was similar to that caused by 50 mg/kg (data not shown).

### 2.2. Tyrosine Hydroxylase (TH) Expression in Striatum and Substantia Nigra

**Figure 3.**
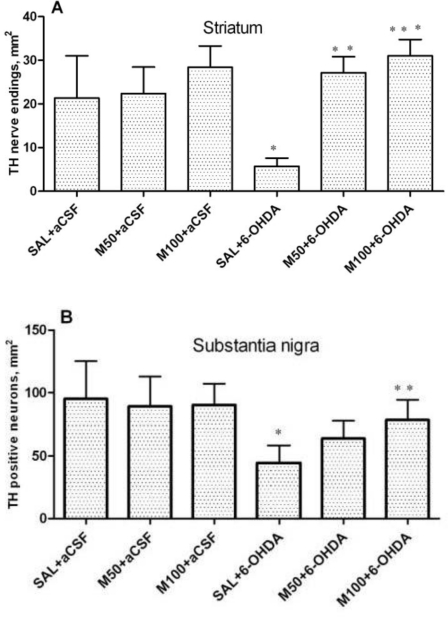
The number of tyrosine hydroxylase (TH)-positive nerve endings in the 6-OHDA-lesioned striatum (**A**) and of TH-positive neurons in the substantia nigra (**B**). Immunohistochemical examination of rat tissue using a TH antibody. Saline (SAL, 1 mL/kg) or mildronate at doses of 50 or 100 mg/kg (M50 and M100, respectively) were administered intraperitoneally for two weeks prior to an injection of 6-OHDA (20 μg) or artificial cerebrospinal fluid (aCSF); 6-OHDA injection in mildronate-treated rats: M50 + 6-OHDA and M100 + 6-OHDA. Striatum: * p = 0.04, SAL + 6-OHDA *vs.* SAL + aCSF; ** p = 0.001, M50 + 6-OHDA *vs.* SAL + 6-OHDA; *** p = 0.0002, M100 + 6-OHDA *vs.* SAL + 6-OHDA; S. nigra: * p = 0.04, SAL + 6-OHDA *vs.* Sal + aCSF; ** p = 0.04, M100 + 6-OHDA *vs.* SAL + 6-OHDA; unpaired t-test. Number of animals per group (n = 8).

**Figure 4.**
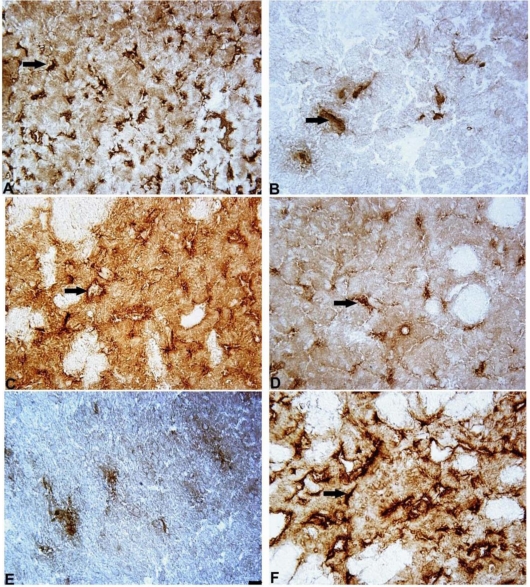
Photomicrograph of tyrosine hydroxylase (TH)-positive neurons in the substantia nigra and TH-positive nerve endings in a 6-OHDA-lesioned striatum. Immunohistochemical staining, magnification × 400. Internal scale bar = 25 μm. S. nigra: (**A**) Saline control (SAL + aCSF); (**B**) SAL + 6-OHDA; the arrow indicates positively stained neurons. (**C**) Striatum: saline control (SAL + aCSF); (**D**) Mildronate at 50 mg/kg + artificial cerebrospinal fluid (M50 + aCSF); (**E**) SAL + 6-OHDA; (**F**) M50 + 6-OHDA; the arrows indicate TH-positive nerve endings.

The expression of TH in the striatum was assessed by counting the number of nerve endings (fibers), and the number of neurons was counted in the SN (Figure 3; photomicrograph in Figure 4). The data demonstrated that 6-OHDA lesions caused a dramatic (five-fold) decrease in TH-positive nerve endings in the lesioned striatum in comparison to the control group (5 ± 2 *vs.* 21 ± 10 nerve endings/per mm^2^, p = 0.03). Mildronate *per se* at the doses of 50 and 100 mg/kg did not influence the density of TH-positive nerve endings. However, the administration of mildronate completely protected against the 6-OHDA-induced decrease in the density of nerve endings (50 mg/kg, 25 ± 4 *vs.* 5 ± 2 nerve endings/mm^2^, 100 mg/kg, 31 ± 3 *vs.* 5 ± 2 nerve fibers/mm^2^, p = 0.001 and p = 0.0002, respectively; Figure 3A).

In the SN, 6-OHDA caused an approximately 2.5-fold decrease in TH levels in comparison to the control group (44 ± 14 *vs.* 95 ± 30 neurons/mm^2^, p = 0.01). Mildronate at a dose of 100 mg/kg protected against the effect of 6-OHDA in this brain structure (78 ± 16 *vs.* 44 ± 14 neurons/mm^2^, p = 0.04; Figure 3B).

### 2.3. The Number of Cells with Intracellular Ubiquitin-Positive Inclusions in Striatum and Substantia Nigra

**Figure 5.**
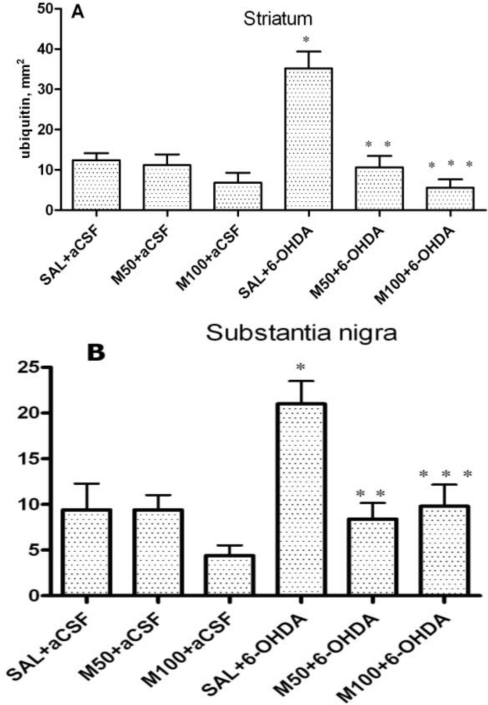
The number of cells that contained ubiquitin-positive inclusions in the 6-OHDA-lesioned striatum (**A**) and substantia nigra (**B**). Immunohistochemical examination of rat tissue using a ubiquitin antibody. Saline at 1 mL/kg (control) and mildronate at doses of 50 and 100 mg/kg were administered intraperitoneally for two weeks prior to the injection of 6-OHDA (20 μg) or artificial cerebrospinal fluid (aCSF); 6-OHDA injection in mildronate-treated rats: M50 + 6-OHDA and M100 + 6-OHDA. Striatum: * p = 0.001, SAL + 6-OHDA *vs.* SAL + aCSF; ** p = 0.001, M50 + 6-OHDA *vs.* SAL + 6-OHDA; S. nigra: *** p = 0.01, M100 + 6-OHDA *vs.* SAL + 6-OHDA; unpaired t-test. Number of animals per group (n = 8).

**Figure 6.**
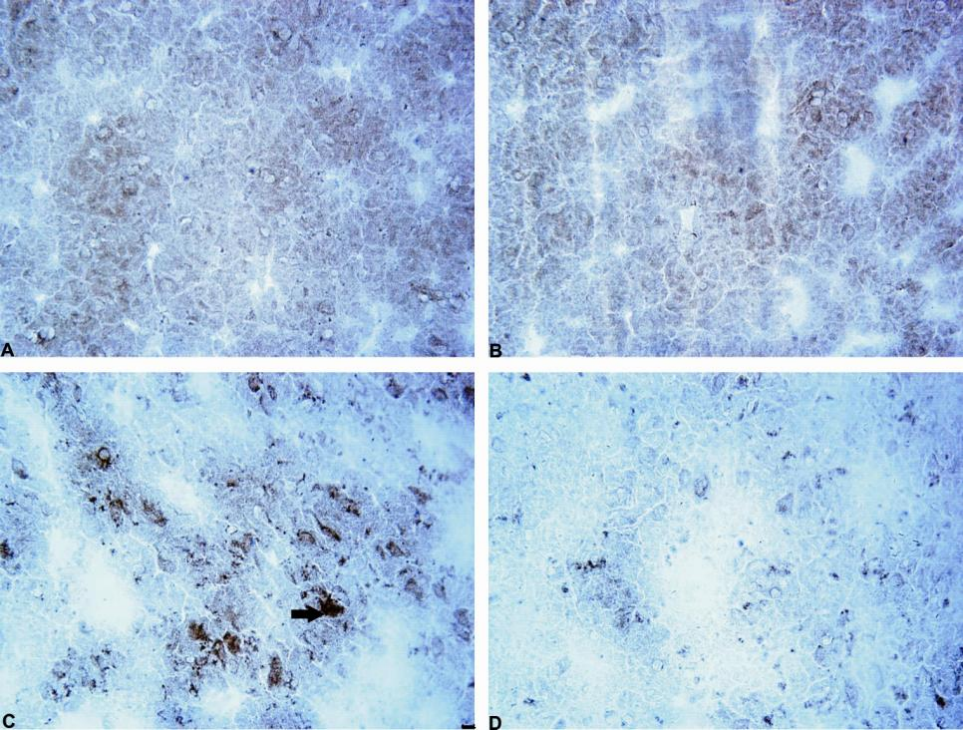
Photomicrograph showing cells containing ubiquitin-positive inclusions in the rat 6-OHDA-lesioned striatum. Immunohistochemical staining, magnification × 400. Internal scale bar = 25 μm. (**A**) Saline control (SAL + aCSF); (**B**) mildronate at 50 mg/kg (M50 + aCSF); (**C**) 6-OHDA (SAL + 6-OHDA); (D) M50 + 6-OHDA; Arrows indicate cells containing ubiquitin-positive inclusions in the 6-OHDA group.

In the saline group, a weak, diffuse ubiquitin positive staining was observed. However, cells that contained ubiquitin-positive inclusions were rare (Figure 5, photomicrograph in Figure 6). In the 6-OHDA group, we observed a three-fold increase in the number of cells with intracellular ubiquitin-positive inclusions in the striatum in comparison to the control (35 ± 4 *vs.* 12 ± 2 cells/mm^2^, p = 0.001, Figure 5A). Mildronate *per se* at a dose of 50 mg/kg did not significantly influence the number of cells that contained ubiquitin-positive inclusions. However, a dose of 100 mg/kg tended to decrease the number of cells that contained these inclusions. Mildronate at both doses, particularly at 100 mg/kg co-administered with 6-OHDA, significantly decreased the 6-OHDA-induced increase in the number of cells with intracellular ubiquitin-positive inclusions (11 ± 3 *vs.* 35 ± 4 cells/mm^2^, p = 0.001, and 6 ± 2 *vs.* 35 ± 4 cells/mm^2^, p = 0.0002).

In the rat SN in the 6-OHDA experimental group, the number of neurons that contained ubiquitin-positive inclusions was significantly increased in comparison to the control (21 ± 2 *vs.* 9 ±3 cells/mm^2^, p = 0.01). Mildronate *per se* at doses of 50 mg/kg and 100 mg/kg did not influence the number of neurons that contained ubiquitin-positive inclusions. However, the administration of mildronate significantly decreased the 6-OHDA-induced increase in the number of cells with intracellular ubiquitin-positive inclusions (8 ± 2 *vs.* 21 ± 2 cells/mm^2^, p = 0.003, and 10 ± 3 *vs.* 21 ±2 cells/mm^2^, p = 0.01; Figure 5B).

### 2.4. Notch-3 Expression in Striatum and Substantia Nigra

**Figure 7.**
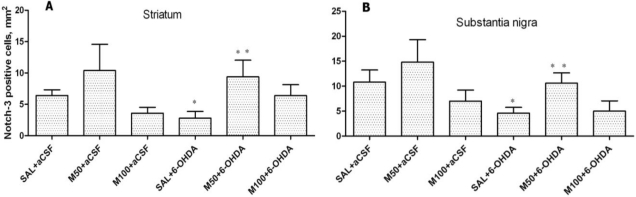
The number of Notch-3-positive cells in the 6-OHDA-lesioned striatum (**A**) and substantia nigra (**B**). Immunohistochemical examination of rat tissue using a Notch-3 antibody. Saline at 1 mL/kg, control (SAL), and mildronate at 50 mg/kg (M50) and 100 mg/kg (M100) were administered intraperitoneally for two weeks prior to the injection of 6-OHDA or artificial cerebrospinal fluid (aCSF); 6-OHDA injection in mildronate-treated rats: M50 + 6-OHDA and M100 + 6-OHDA. Striatum: * p = 0.03, SAL + 6-OHDA *vs.* SAL + aCSF; •p = 0.04, M50 + 6-OHDA *vs.* SAL + 6-OHDA S. nigra: * p = 0.049, SAL + 6-OHDA *vs.* SAL + aCSF; •p = 0.03, M50 + 6-OHDA *vs.* SAL + 6-OHDA; unpaired t-test. Number of animals per group (n = 8).

**Figure 8.**
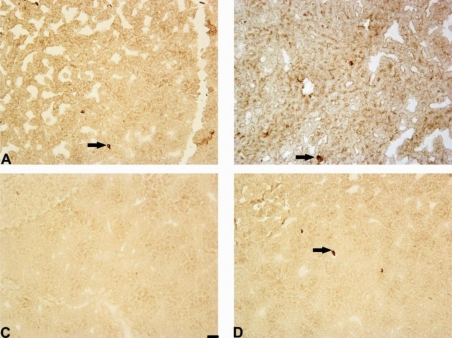
Photomicrograph of Notch-3-positive cells in the striatum. Immunohistochemical staining, magnification × 200. Internal scale bars = 50 μm. (**A**) Saline; (**B**) mildronate at 50 mg/kg; (**C**) 6-OHDA; (**D**) M50 + 6-OHDA. The administration of 6-OHDA decreased the number of Notch-3-positive cells in the rat striatum, but the co-administration of mildronate at 50 mg/kg and 6-OHDA increased the number of Notch-3-positive cells to values similar to those observed in controls. Arrows indicate positively stained cells.

The administration of 6-OHDA decreased the number of Notch-3-positive cells in the rat striatum in comparison to the control group (3 ± 1 *vs.* 6 ± 1 cells/mm^2^, p = 0.04; Figures 7A and 8). Mildronate at doses of 50 mg/kg and 100 mg/kg tended to increase and decrease the number of Notch-3-positive cells, respectively. Mildronate (50 mg/kg) co-administered with 6-OHDA increased the number of Notch-3-positive cells in comparison to the 6-OHDA group (9 ± 3 *vs.* 3 ± 1 cells/mm^2^, p = 0.045). The 100 mg/kg dose of mildronate tended to increase the number of Notch-3-positive cells compared to 6-OHDA group.

In the rat SN, 6-OHDA decreased the number of Notch-3-positive cells in comparison to the control group (5 ± 1 *vs.* 11 ± 2 cells/mm^2^, p = 0.05). Mildronate at 50 mg/kg, but not at 100 mg/kg, tended to increase the number of Notch-3-positive cells. Mildronate at 50 mg/kg, but not at 100 mg/kg, co-administered with 6-OHDA increased the number of Notch-3-positive cells in comparison to the 6-OHDA group (11 ± 2 *vs.* 5 ± 1 cells/mm^2^, p = 0.03; Figures 7B and 8).

### 2.5. GFAP Expression in Striatum and Substantia Nigra

**Figure 9.**
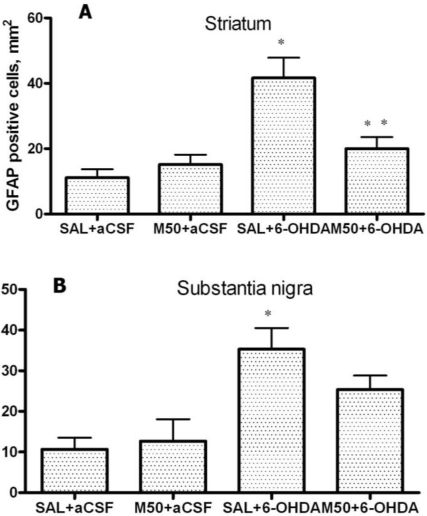
(**A**) The number of GFAP-positive astrocytes in the 6-OHDA-lesioned striatum; and (**B**) substantia nigra. Immunohistochemical examination of rat tissue using a GFAP antibody. Saline (SAL, 1 mL/kg) and mildronate at 50 mg/kg (M50) and 100 mg/kg (M100) were administered intraperitoneally two weeks prior to the injection of 6-OHDA or artificial cerebrospinal fluid (aCSF); 6-OHDA injection in mildronate-treated rats: M50 + 6-OHDA and M100 + 6-OHDA. Striatum: * p = 0.001, SAL + 6-OHDA *vs.* SAL + aCSF; ** p = 0.01, M50 + 6-OHDA *vs.* SAL + 6-OHDA; S. nigra: * p = 0.002, SAL + 6-OHDA *vs.* SAL + aCSF; unpaired t-test. Number of animals per group (n = 8).

Figure 9A shows that in the 6-OHDA-lesioned striatum, a four-fold increase in the number of GFAP-positive glial cells was observed in comparison to the control group (42 ± 6 *vs.* 11 ±3 cells/mm^2^, p = 0.001). Mildronate alone at doses of 50 and 100 mg/kg did not significantly influence GFAP expression. However, in the mildronate-treated rats (50 mg/kg), a two-fold decrease in 6-OHDA-induced GFAP overexpression was observed (20 ± 4 *vs.* 42 ± 6 cells/mm^2^, p = 0.01).

The administration of 6-OHDA also caused GFAP overexpression in the SN in comparison to the control group (35 ± 5 *vs.* 11 ± 3 cells/mm^2^, p = 0.002; Figure 9B). The co-administration of mildronate at 50 mg/kg and 6-OHDA showed a tendency to decrease the number of GFAP-positive glial cells. Mildronate at 100 mg/kg was not tested in this study.

**Figure 10.**
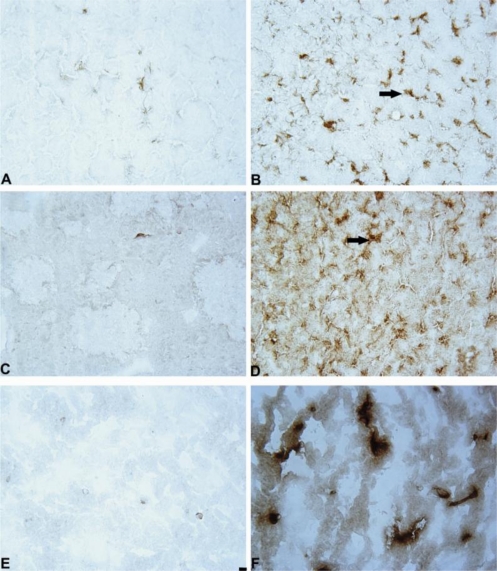
Photomicrograph of GFAP, iNOS positive cells in the 6-OHDA-lesioned striatum and IBA-1 positive cells in S.nigra. Immunohistochemical staining, magnification × 400. Internal scale bar = 25 μm. (**A**) GFAP: SAL + aCSF; (**B**) GFAP: SAL + 6-OHDA; (**C**) iNOS: SAL + aCSF; (**D**) iNOS: SAL + 6-OHDA; (**E**) IBA-1: SAL + aCSF; (**F**) IBA-1: SAL + 6-OHDA. Arrows indicate positively stained cells.

Figure 10A, B shows the immunohistochemical staining for GFAP-positive astrocytes.

### 2.6. IBA-1-Expression in Striatum and Substantia Nigra

**Figure 11.**
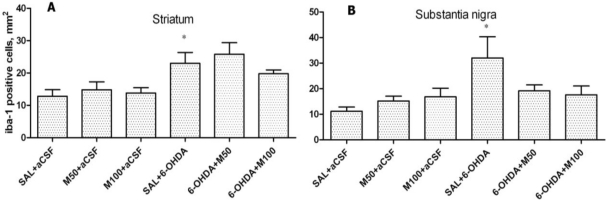
The number of IBA-1-positive cells in the 6-OHDA-lesioned striatum (**A**) and substantia nigra (**B**). Immunohistochemical examination of rat tissue using an IBA-1 antibody. Saline (SAL, 1 mL/kg) and mildronate at doses of 50 mg/kg and 100 mg/kg (M50 and M100, respectively) were administered intraperitoneally two weeks before the injection of 6-OHDA or artificial cerebrospinal fluid (aCSF); 6-OHDA injection in mildronate-treated rats: M50 + 6-OHDA and M100 + 6-OHDA; striatum: * p = 0.03, SAL + 6-OHDA *vs.* SAL + aCSF; s. nigra: * p = 0.04, SAL + 6-OHDA *vs.* SAL + aCSF; unpaired t-test. Number of animals per group (n = 8).

An increase in the number of IBA-1-positive cells was found in the 6-OHDA-lesioned striatum in comparison to the control group (23 ± 3 *vs.* 12 ± 2 cells/mm^2^, p = 0.03; Figure 11A). Mildronate at 50 and 100 mg/kg did not significantly influence IBA-1 expression in comparison to the control group. The co-administration of mildronate at 50 mg/kg and 6-OHDA tended to increase the number of IBA-1-positive cells. However, a dose of 100 mg/kg mildronate tended to decrease the number of IBA-1-positive cells.

The administration of 6-OHDA significantly increased the number of IBA-1-positive cells in the SN in comparison to the control group (32 ± 8 *vs.* 11 ± 2 cells/mm^2^, p = 0.04; Figure 11). Mildronate *per se* at doses of 50 and 100 mg/kg did not significantly influence the expression of IBA-1, but the co-administration of mildronate and 6-OHDA showed a tendency to decrease the number of IBA-1-positive cells (18 ± 2 *vs.* 32 ± 8, p = 0.14; and 17 ± 2 *vs.* 32 ± 8, p = 0.15, respectively).

Figure 10E, F shows the immunohistochemical staining for IBA-1-positive microglial cells.

### 2.7. iNOS Expression in Striatum and Substantia Nigra

**Figure 12.**
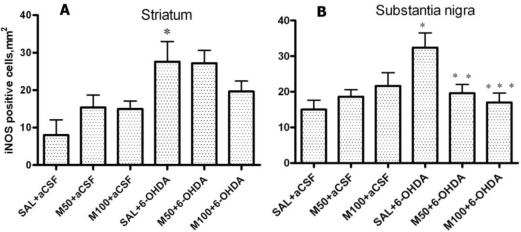
The number of iNOS-positive cells in the 6-OHDA-lesioned striatum (**A**) and substantia nigra (**B**). Immunohistochemical examination of rat tissue using an iNOS antibody. Saline (SAL, 1 mL/kg) and mildronate at doses of 50 mg/kg and 100 mg/kg (M50 and M100, respectively) were administered intraperitoneally two weeks prior to the injection of 6-OHDA or artificial cerebrospinal fluid (aCSF); 6-OHDA injection in mildronate-treated rats: M50 + 6-OHDA and M100 + 6-OHDA; striatum: * p = 0.02, SAL + 6-OHDA *vs.* SAL + aCSF; s. nigra: * p = 0.007, SAL + 6-OHDA *vs.* SAL + aCSF; ** p = 0.045, M50 + 6-OHDA *vs.* SAL + 6-OHDA; *** p = 0.04, M100 + 6-OHDA *vs.* SAL + 6-OHDA; unpaired t-test. Number of animals per group (n = 8).

The administration of 6-OHDA caused a considerable increase in iNOS expression in the lesioned striatum and SN (Figure 12A, B). Mildronate alone did not influence iNOS expression. The co-administration of mildronate at a dose of 50 mg/kg and 6-OHDA significantly decreased the number of iNOS-positive cells in the SN (Figure 12B).

Figure 10C, D show the immunohistochemical staining for iNOS-positive cells.

## 3. Discussion

Recent new data demonstrate that macroglial and microglial cells contribute considerably to homeostasis in the brain [25]. Therefore, novel therapeutic strategies for the treatment of PD can be rationally focused on both neuronal and glial processes. Mitochondria, which play a critical role in the aetiology of PD [39], can be considered one of the most essential targets that are common to both neurons and glial cells. The oxidation rates of various substrates and the acceptor control ratios do not differ appreciably between neuronal and glial cell mitochondria [40]. In this context, mitochondria are vulnerable to the effects of 6-OHDA-induced cell death [41]. Moreover, 6-OHDA has been proposed to be a putative endogenous neurotoxic factor in the pathogenesis of PD because it can be generated from dopamine via non-enzymatic hydroxylation processes in the presence of Fe^2+^ and H_2_O_2_ [42].

We suggest that a small molecule, mildronate, might function as a suitable “interface molecule” between neuronal and glial systems and correct the imbalances that are present in the rat model of PD. This assumption was based on our previous findings that demonstrated the protective effects of mildronate at the level of the mitochondrial respiratory chain complex I [38] and the ability of mildronate to exert anti-inflammatory and anti-apoptotic effects in a rat model of neurotoxicity generated with the mitochondria-compromising drug azidothymidine [37]. In the present study, we assessed numerous markers of impaired neuronal and glial cell functions in the nigro-striatal system.

In our study the rotations behavior test showed that both doses of mildronate decreased the apomorphine rotations in 6-OHDA-treated rats, particularly on day 21, which indicated that mildronate attenuated behavioral disturbances in a PD model.

An immunohistochemical examination of brain tissue *ex vivo* demonstrated that 6-OHDA-induced lesions were associated with a massive loss of neuronal dopamine, a dramatic (five-fold) decrease in the number of TH-positive nerve endings in the lesioned striatum and an approximately 2.5-fold decrease in the number of TH-positive neurons in the SN in comparison to the control group (saline + aCSF). The administration of mildronate at a dose of 50 mg/kg provided complete protection against the 6-OHDA-induced degeneration of TH-positive nerve endings in the lesioned striatum. In addition, mildronate at 100 mg/kg protected against the loss of TH-positive dopaminergic neurons in the SN.

Our results are consistent with previous findings demonstrating that 6-OHDA decreased both the number of TH-positive nerve endings in the striatum and the number of TH dopaminergic neurons in the SN [23]. Therefore, our data demonstrate the neuroprotective properties of mildronate against a 6-OHDA-induced loss of TH expression. These findings raise the following question: what are the crucial mechanisms responsible for this protective action of mildronate?

Our results demonstrated the ability of mildronate to completely prevent (to control level) the 6-OHDA-induced formation of ubiquitin intracellular inclusions in both the rat striatum and SN. The increased level of unconjugated ubiquitin and the formation of intracellular ubiquitin inclusions in the 6-OHDA dopamine-depleted striatum observed in the present study are consistent with the findings presented in the literature [43]. The effect of mildronate leads us to suggest that it is capable of regulating the ubiquitin proteasome pathway, probably by activating the proteasome and/or enhancing ubiquitination and thereby promoting protein degradation. An explanation of this effect may be the ability of mildronate to protect cells at the level of the mitochondria [38] because disturbances in ubiquitin production depend largely on oxidative stress-induced impairments in glucolysis and mitochondrial respiration [44]. The central role of the ubiquitin-proteasome system in regulating the degradation of cellular proteins under different physiological conditions has been increasingly recognized.

The accumulation of misfolded proteins in the cells is involved in the pathogenesis of many neurodegenerative diseases, such as Parkinson’s disease (PD), Alzheimer’s disease (AD) and Huntington’s disease (HD) [19,20,43].

Our results have demonstrated that 6-OHDA decreased the number of Notch-3 positive cells in rat striatum and substantia nigra. However, mildronate at a dose of 50 mg/kg coadministrated with 6-OHDA increased the number of Notch-3-positive cells close to control values.

The Notch family of receptors and ligands plays an important role in the determination of cell fate, vasculogenesis and organogenesis, and the activation of Notch-1, 2 and 3 receptors has multiple roles during CNS development, particularly during gliogenesis [45]. Furthermore, Notch-3 is a marker of progenitor cells and appears to couple the Notch pathway to the regulation of cell growth [46]. The presence of endogenous progenitor cells in the adult mammalian brain presents an attractive alternative to the available therapeutic options for the treatment of PD [32]. To date, we have not found any data showing Notch-3 expression in PD or PD animal models. The effect of mildronate on Notch-3 expression is difficult to explain. However, we think that it is an interesting phenomenon and suggest that mildronate stimulates the adult striatal progenitor cell population.

In addition, we demonstrated that 6-OHDA increased the number of GFAP-positive astrocytes in striatum and SN. This finding is consistent with previous data that demonstrated an increase in the levels of GFAP in 6-OHDA-lesioned structures [32,46]. The administration of mildronate at a dose of 50 mg/kg decreased the number of GFAP-positive astrocytes to a level similar to that detected in the control. We consider this phenomenon to be a positive effect of mildronate because it coincided with the normalization of TH expression, which affects cell survival. A similar normalization effect was observed in studies investigating the novel anti-parkinsonian agent zonisamide, which was found to attenuate MPTP-induced neurotoxicity in mice [47].

There are different hypotheses concerning the mechanisms underlying reactive astrogliosis [48,49]. On the one hand, activated astrocytes may stimulate microglial cells, which induce dopamine cell sprouting via the synthesis of neurotrophic factors [50]. Therefore, astrocytes can act as stem cells during adult neurogenesis [51]. On the other hand, sustained inflammatory astrogliosis may have deteriorating effects after brain injuries and in neurodegenerative and demyelinating diseases [51].

In the present study, we also assessed the microglial marker IBA-1, which is specifically expressed in macrophages/microglia and is upregulated when these cells are activated [52]. We found that the expression of IBA-1 was increased by two-fold in the 6-OHDA-lesioned striatum and by 2.5-fold in the SN in comparison to the control group. These findings are consistent with the results described in the literature, which show that 6-OHDA causes neuroinflammation and microglial activation [21] by increasing the expression of proinflammatory cytokines [52]. Interestingly, in the 6-OHDA-lesioned SN, microglial activation precedes dopamine neuronal loss [21,22]. Microglial activation is important because reactive microglia may have a destructive influence on neurons. Conversely, activated microglia may stimulate the production of substances with neurotrophic properties [50]. In the present study, mildronate did not affect the level of IBA-1 in the 6-OHDA-lesioned striatum, but in the SN, it showed a tendency to decrease the expression of IBA-1. Because IBA-1 activation coincided with an increase in TH and Notch-3 expression in the presence of mildronate, microglial activation may have a beneficial role in cell survival.

Finally, in the 6-OHDA-lesioned striatum and SN, we detected a considerable increase in the expression of the inflammatory marker iNOS, which indicated a manifestation of neuroinflammatory processes. Similarly, iNOS overexpression has been previously observed in the 6-OHDA model [53], which indicates that iNOS induction plays an important role in the initial phase of neurodegeneration. The administration of mildronate at a dose of 50 mg/kg decreased the expression of iNOS in the SN by approximately two-fold, which indicates that mildronate possesses anti-inflammatory activity.

In summary, our data suggest that mildronate is capable of regulating many endogenous molecules that are involved in cell survival processes. The most important findings in the present study include the complete prevention of the loss of TH in the 6-OHDA-lesioned striatum and the decreased number of ubiquitin-positive intracellular inclusions. Furthermore, mildronate decreased GFAP and iNOS expression but stimulated Notch-3 expression. These data indicate that mildronate controls an array of cell proteins that participate in multi-faceted homeostatic mechanisms and provide neuroprotection in PD.

## 4. Experimental Section

### 4.1. Materials and Methods

*Animals.* Male Wistar rats were obtained from the Laboratory of Experimental Animals, Riga Stradins University, Riga, Latvia. Each animal weighed 230–250 g at the beginning of each experiment. All of the experimental procedures were performed in accordance with the guidelines of Directive 86/609/EEC “European Convention for the Protection of Vertebrate Animals Used for Experimental and Other Scientific Purposes” (1986) and were approved by the Animal Ethics Committee of the Food and Veterinary Service (Riga, Latvia).

*Drugs.* Mildronate [3-(2,2,2-trimethylhydrazinium) propionate dihydrate] was obtained from the Joint Stock Company “Grindex” (Riga, Latvia), dissolved in saline and prepared as a 2% stock solution. The 6-hydroxydopamine (6-OHDA) was obtained from *Sigma-Aldrich* (St. Louis, MO, USA), dissolved in 0.2% ascorbic acid solution and prepared as a stock solution at 20 μg/3 μL. This solution was made fresh and protected from light exposure. Apomorphine from *Sigma-Aldrich* (St. Louis, MO, USA) was dissolved in 0.1% ascorbic acid solution. Ketamine, xylazine and imipramine were purchased from *Sigma-Aldrich* (St. Louis, MO, USA).

The following antibodies were used: rabbit polyclonal IBA-1 (Wako Chemicals, USA); rabbit polyclonal TH (Millipore, USA); rabbit polyclonal Notch-3 (Santa Cruz Biotechnology, USA); rabbit polyclonal ubiquitin (Santa Cruz Biotechnology, USA); rabbit polyclonal GFAP (DAKO, Denmark); and rabbit polyclonal iNOS (Abcam, UK).

The EnVision detection kit, peroxidase-conjugated polyclonal goat anti-rabbit IgG and 3,3-diaminobenzidine (DAB) were purchased from DAKO, Denmark.

### 4.2. Experimental Design

The rats were adapted to the experimental conditions and divided into six groups (eight animals per group). Mildronate or saline (control) in a volume of 1 mL/kg was administered intraperitoneally (i.p.) every day for 14 days at doses of 50 or 100 mg/kg. On day 15, the neurotoxin 6-OHDA was administered into the right corpus striatum at a concentration of 20 μg/3 μL. The control group received artificial cerebrospinal fluid (aCSF).

Groups:

Group 1: Saline (i.p.) for two weeks followed by aCSF (intrastriatal)(corpus striatum).
Group 2: Mildronate, 50 mg/kg i.p. for two weeks followed by aCSF (intrastriatal).
Group 3: Mildronate, 100 mg/kg i.p. for two weeks followed by aCSF (intrastriatal).
Group 4: Saline i.p. for two weeks followed by 6-OHDA (intrastriatal).
Group 5: Mildronate, 50 mg/kg i.p. for two weeks followed by 6-OHDA (intrastriatal).
Group 6: Mildronate, 100 mg/kg i.p. for two weeks followed by 6-OHDA (intrastriatal).

### 4.3. Surgical Procedures

After the intraperitoneal administration of mildronate or saline for two weeks, the rats were anesthetized (ketamine 75 mg/kg + xylazine 10 mg/kg) and placed in a *stereotaxic* frame (Stoelting Inc., USA). Thirty minutes before the induction of general anesthesia, the rats received imipramine (at a dose of 20 mg/kg) to protect adrenergic neurons against the development of 6-OHDA-induced lesions.

Anesthetized rats received 20 μg/3 μL 6-OHDA solution or aCSF in the right striatum using a *Stoelting* microinjector. The injection rate was 1 μL/min, and the cannula was maintained in the delivery position for an additional 3 min prior to its slow retraction. Coordinates (according to *Paxinos & Watson*): AP + 1.2; LM + 2.5; DV − 5.0 mm from the bregma.

### 4.4. Rotational Behavior

On days 14, 21 and 28 after surgery and the administration of 6-OHDA, 0.2 mg/kg apomorphine (dopamine receptor agonist) was injected subcutaneously, and the number of asymmetric (contralateral) rotations for each animal was monitored over 30 min.

### 4.5. Brain Tissue Processing

After termination of the rotational behavior test (on day 28), the rats were anesthetized with ketamine (150 mg/kg) and perfused through the ascending aorta with 50 mL of isotonic saline followed by 250 mL of 4% paraformaldehyde in 0.1 M phosphate buffer (pH 7.4). The brains were removed and fixed at −80 °C.

The brain tissue was cut into 10-μm-thick sections using a cryostat at −20 °C *(Leica CM1850*, *Leica Microsystems, Germany*), and 24 sections each from the *corpus striatum* (striatum) and *substantia nigra* (s. nigra) were obtained.

The sections were transferred to polylysine-coated slides (3 sections on each slide). The slides were air-dried for 15 minutes and then immersed in ice-cold acetone for 15 min and air-dried for 2 hours. The slides were wrapped in aluminum foil and stored at −20 °C.

### 4.6. Immunohistochemical Examination of the Brain Tissue

Ten-micron-thick tissue sections were stained to visualize the cells that were positive for TH, ubiquitin, Notch-3, IBA-1, GFAP and iNOS, according to a previously described immunohistochemical procedure [40].

Briefly, endogenous peroxidase activity was blocked with 3.0% H_2_O_2_ for 10 min. Nonspecific primary antibody binding was blocked by incubating the slides with normal horse serum. These slides were incubated with rabbit polyclonal IBA-1 (Wako Chemicals, dilution at 1:500), rabbit polyclonal TH (Millipore, 1:400), or Notch-3 (Santa Cruz Biotechnology, 1:100), ubiquitin (1:100), or iNOS (Abcam, 1:100) overnight at 4 °C. The slides were incubated with rabbit polyclonal GFAP (DAKO, 1:500) for one hour at room temperature.

The rabbit polyclonal ubiquitin antibody was obtained from Santa Cruz Biotechnology (sc9133, clone FL-76). The epitope of the ubiquitin antibody corresponded to amino acids 1–76 and represented a full-length ubiquitin molecule of human origin. This antibody labels human, mouse and rat ubiquitin and polyubiquitin.

For TH, GFAP and iNOS immunostaining, the detection of bound antibody was performed using EnVision reagent (Dako, Denmark). To detect the expression of Notch-3, IBA-1 and ubiquitin, the slides were incubated with peroxidase-conjugated polyclonal goat anti-rabbit IgG for one hour.

The immunoperoxidase colour reaction was developed by incubating the slides with diaminobenzidine (5 min). A negative control without primary antibody was included in each experiment.

### 4.7. Imaging and Quantitation of Fibers and Cells

*Quantitation of cells in the striatum.* Tyrosine hydroxylase-immunopositive fibers were counted bilaterally in six independent sections each from all of the experimental groups. The total number of TH-positive nerve fibers in the striatum was quantified using a ×40 objective. The region of interest was captured using a Motic digital camera (Motic, China) mounted on a microscope (Motic BA400) using Motic Image Advanced 3.2 software. Whole striatal sections were captured and analyzed. The results are expressed as fibers per square millimeter.

A similar approach was utilized to count the number of GFAP, iNOS and IBA-1-positive cells. In addition, the number of cells that contained ubiquitin-positive inclusions was determined.

*Quantitation of cells in the substantia nigra*. To quantify the number of tyrosine hydroxylase (TH)-positive neurons, the positive cells were counted using a Motic BA400 microscope connected to a camera at a magnification of ×40. TH-positive neurons were counted in both the ipsilateral and contralateral hemispheres of the brain in each of the stereotaxic regions of the SN and adjacent ventral tegmental area (VTA) area to ensure complete sampling of the entire structure. The stereotaxic region in each section was determined by examining the shape and distribution of TH-positive neurons at a low magnification (4×). The substantia nigra (SN) was delineated from the VTA by reference to the specific anatomical landmark of the third cranial nerve rootlet. To minimize bias, all of the operators were assessed for counting consistency at each stereotaxic level of the SN prior to performing the cell counting. Intact cells with a nucleus, clear cytoplasm and axonal processes were included in the cell counts, and cells that appeared deformed and that lacked clear axonal processes were excluded from the cell counts. For each animal, six consecutive sections were counted at each stereotaxic level, and the results were pooled to obtain a total mean cell count for the ipsilateral and contralateral hemispheres in each group. To ensure an unbiased cell count, all of the operators were blinded to the analyzed group.

A similar approach was utilized to count the numbers of GFAP, iNOS and IBA-1-positive cells. In addition, the number of cells that contained ubiquitin-positive inclusions was counted.

### 4.8. Statistics

For the statistical analysis, GraphPad Prism 5 software was used. The results are expressed as the mean ± SEM, and the level of significance was p < 0.05 (unpaired t-test).

## 5. Conclusions

Mildronate, a small molecule of the aza-butyrobetaine class, shows neuroprotective activity in a rat model of Parkinson’s disease by preventing the alterations of the neuronal-glial pathways in the 6-OHDA-lesioned striatum and substantia nigra. We suggest that mildronate has a neuroprotective effect and recommend its use during the early stages of PD to delay or halt the progression of this neurodegenerative disease.
